# Molecular and Cellular Signaling Pathways of the Effects of Hypoxia and Hypercapnia on the Mechanisms of Neuroinflammation

**DOI:** 10.3390/ijms27125579

**Published:** 2026-06-20

**Authors:** Pavel A. Chekulaev, Georgy M. Zembatov, Eugenia D. Namiot, Tatiana M. Alekseeva, Ivan K. Ternovykh, Zaripat S. Manasova, Vladimir P. Kulikov, Natalia S. Andriutsa, Pavel P. Tregub

**Affiliations:** 1 Brain Science Institute, Russian Center of Neurology and Neurosciences, 125367 Moscow, Russia; 2 Department of Pathophysiology, I.M. Sechenov First Moscow State Medical University, 119991 Moscow, Russia; 3 V.A. Almazov National Medical Research Center, 197341 St. Petersburg, Russia; 4 Department of Ultrasound and Functional Diagnostics, Altay State Medical University, 656040 Barnaul, Russia; 5 Scientific and Educational Resource Center “Innovative Technologies of Immunophenotyping, Digital Spatial Profiling and Ultrastructural Analysis”, RUDN University, 117198 Moscow, Russia

**Keywords:** hypercapnic hypoxia, hypercapnia, hypoxia, neuroinflammation, microglia

## Abstract

Recovery after an ischemic stroke depends not only on neuronal survival but also on inflammatory mechanisms that determine secondary injury and reparative plasticity. This review summarizes the evidence on hypoxic conditioning, permissive hypercapnia, and their combined application as modulators of neuroinflammation and neurorehabilitation. This review does not aim to describe the fundamental mechanisms of neuroinflammation, but rather to examine how hypoxia, hypercapnia, and their interaction provide potential targets for its modulation. Prolonged or severe hypoxia exacerbates neuroinflammation through NF-κB activation, NLRP3 inflammasome signaling, pro-inflammatory cytokine production, and microglial activation. In contrast, controlled intermittent hypoxia in pre-/postconditioning protocols suppresses inflammatory processes, promotes reparative microglial phenotypes, activates PI3K/Akt-dependent survival pathways, and modulates the fractalkine/CX3CR1 axis. Permissive hypercapnia also has context-dependent immunomodulatory properties: moderate exposure may reduce NF-κB-driven inflammation, oxidative damage, apoptosis, and blood–brain barrier disruption, whereas prolonged hypercapnia, especially with hypoxemia, may enhance inflammasome activation and microglial reactivity. Therefore, combined intermittent hypercapnic hypoxia may act as a therapeutic stimulus integrating anti-inflammatory, cytoprotective, barrier-stabilizing, and neuroplastic mechanisms. Clinical evidence regarding ischemic stroke and cerebral palsy is encouraging but limited. Future studies should determine optimal gas exposure protocols, precisely define the mechanisms underlying the anti-inflammatory effects, and establish whether pharmacological potentiation using modulators of the NLRP3, PI3K/Akt, BDNF/TrkB, and JNK signaling pathways is feasible.

## 1. Introduction

The search for effective neurorehabilitation approaches for ischemic stroke is one of the most urgent tasks in clinical and experimental neuroscience. Hypoxic breathing training is a promising strategy for treating neurological diseases, including ischemic stroke [1]. Several experimental studies have demonstrated the effectiveness of intermittent hypoxic exposure as a therapeutic tool for various pathologies of the nervous system, including perinatal CNS and ischemic brain injuries [2,3,4]. At the same time, over the past decade, an increasing number of studies have focused on the therapeutic efficacy of permissive hypercapnia, showing that inhalation of carbon dioxide at a safe concentration has a protective effect on the brain under ischemic/hypoxic injury conditions [5,6].

One promising approach is exposure to hypercapnic hypoxia, a combination of normobaric hypoxia and permissive hypercapnia [7]. Experimental data indicate that combined exposure to hypoxia and hypercapnia produces a more pronounced neuroprotective effect than when they are applied separately [8,9]. Hypercapnic hypoxia has demonstrated high efficacy as a means of stimulating reparative and adaptive processes during neurorehabilitation after an ischemic stroke [10]. Thus, regular sessions of hypercapnic hypoxia during the recovery period after a stroke have been shown to accelerate regenerative processes, improve motor function, and reduce signs of neurological deficit both under experimental in vivo conditions in rats [8] and in clinical settings in patients 24–72 h after an ischemic stroke [10].

Against this backdrop, there is growing interest in studying the neurorehabilitation potential of hypercapnic hypoxia as an independent therapeutic intervention aimed at modulating adaptive and metabolic processes after an ischemic injury. A range of mechanisms underlying the neuroprotective efficacy of combined hypercapnia and hypoxia exposure preceding experimental ischemic injury have been studied [11]. Some signaling systems are associated with the predominant influence of the hypoxic stimulus, including HIF-1α and A1 receptors, while others, including NF-κB, antioxidant activity, inhibition of apoptosis, and maintenance of selective blood–brain barrier permeability, are mainly modulated by hypercapnia [11]. Most molecular and cellular mechanisms involved in the development of cerebral ischemic tolerance—including ATP-dependent potassium channels, chaperones, endoplasmic reticulum stress, and reprogramming of mitochondrial metabolism—are determined by the contribution of both excess carbon dioxide and oxygen deficiency [11].

However, the roles of inflammation-modulating signaling pathways in the mechanisms of neuroprotection and neuroplasticity after exposure to hypercapnic hypoxia remain insufficiently studied. At the same time, inflammation plays one of the leading roles in the pathogenesis of many neurological disorders, including ischemic injuries [12,13].

The aim of this review is not to describe the mechanisms of neuroinflammation per se, but rather to examine the potential points of modulation of neuroinflammation by hypoxia, hypercapnia, and their combined action. Potential synergistic mechanisms of neuroprotective efficacy that arise when these factors are combined, as well as the prospects for the development of new strategies for pharmacological modulation of the inflammatory process, are also considered.

To more precisely distinguish the contributions of individual respiratory stimuli, this review first examines the effects of hypoxia and permissive hypercapnia under isolated exposure conditions. Then, it analyzes their combined action under hypercapnic hypoxia. This sequential presentation allows for the separate evaluation of oxygen-dependent, CO_2_-dependent, and potentially synergistic components of the response. This distinction is particularly important when analyzing neuroinflammation because the net biological effect is determined by gas concentrations, exposure duration, acid–base status, tissue context, and the nature of the primary injury.

## 2. Materials and Methods

A systematic search of the relevant literature published from 1 January 2000 to 30 April 2026, was conducted. Four authors independently performed the search for scientific publications in the PubMed and Google Scholar databases using the following queries: (“hypoxic preconditioning” OR “hypoxia preconditioning” OR “hypoxic postconditioning” OR “hypoxia postconditioning” OR “hypoxia”) AND (neuroinflammation OR neuroinflammatory response OR brain inflammation OR microglial activation) and (“hypercapnic preconditioning” OR “hypoxia-hypercapnia preconditioning” OR “hypercapnic postconditioning” OR “hypoxia-hypercapnia postconditioning” OR “hypercapnia”) AND (neuroinflammation OR neuroinflammatory response OR brain inflammation). After independent screening, all potentially relevant articles were discussed collegially, and the authors jointly decided to include data from each cited work.

## 3. Effect of Hypoxic Conditioning on the Mechanism of Neuroinflammation

Hypoxia is one of the major factors contributing to cell and tissue damage in a wide range of pathologies, including ischemic stroke, chronic coronary syndrome, and obstructive sleep apnea [14,15,16]. At the same time, the severity and duration of hypoxia determine the balance between its protective, conditioning, and damaging effects [15]. Prolonged hypoxia and critically low oxygen levels—observed, for example, in models of obstructive sleep apnea—may lead to tissue damage and an increase in signs of neuroinflammation even after exposure for a moderate duration. Thus, intermittent hypoxia (5% O_2_) was found to aggravate intracerebral hemorrhage and increase the secretion of IL-1β, IL-6, TNF-α, and NF-κB in the hippocampus in an animal model of obstructive sleep apnea [16]. Additionally, intermittent hypoxia induced by 8 h inhalation of a gas mixture containing 10% O_2_ led to impaired performance in cognitive tests and increased expression of the pro-inflammatory molecules IL-1β, TNF-α, and NF-κB [17]. Chronic hypoxia at an O_2_ level of 1% induced microglial activation and increased the expression of the enzymes iNOS and COX-2 [18]. Similar findings have also been obtained in other studies [19,20,21]. Thus, prolonged or severe hypoxia leads to an intensification of neuroinflammatory processes.

A number of studies have shown evidence of a possible anti-inflammatory response to hypoxia when it is used as a pre- or postconditioning stimulus. For example, after modeling transient global cerebral ischemia, Lu et al. [22] showed that hypoxic preconditioning through inhalation of a gas mixture containing 8% O_2_ for 30 min induces changes that directly affect neuroinflammation [22]. Thus, hypoxic preconditioning reduced the level of phosphorylated NF-κB and prevented its translocation to the nucleus while concurrently increasing the level of IκB, which inhibits this process through phosphorylation followed by ubiquitination and proteolysis [23]. This resulted in a decrease in the level of the NLRP3 inflammasome. NF-κB is well known as one of the most important regulators of inflammation. It ensures the expression of a wide range of pro-inflammatory molecules and cytokines [24]. In addition, NF-κB is one of the factors contributing to the development of neuroinflammation in ischemia/reperfusion brain injuries [24].

The effect of hypoxic preconditioning in a middle-cerebral-artery occlusion model (MCAo) on NLRP3 inflammasome formation was also confirmed in another study, where hypoxic preconditioning was induced by temporarily stopping the flow of air into an animal chamber [25]. These results suggest that hypoxic preconditioning may reduce pyroptotic activity after ischemia. In addition, hypoxic exposure was associated with reduced levels of MLKL, which is involved in NF-κB activation and the development of necroptosis [22]. It has also been shown that hypoxic preconditioning reduces IL-1R1 expression, leading to decreased MLKL expression and reduced translocation of MLKL to the membrane, in turn potentially preventing cell death resulting from necroptosis [26].

It can be hypothesized that the anti-inflammatory effect of hypoxia may be partially mediated by reduced activity of NF-κB and NLRP3 through activation of IκB, MLKL, and IL-1R1—key regulators of inflammation and necroptosis [22,23,24,25,26].

In contemporary studies, two conventional phenotypes of activated microglia are often distinguished: M1, which is pro-inflammatory, and M2, which is reparative/anti-inflammatory [27,28]. Nevertheless, it is worth noting that experts in the fields of neuroimmunology and microglial research are increasingly discussing the limitations of this dichotomy. Existing M1 and M2 phenotype markers can be co-expressed on a single cell, making data interpretation challenging. In accordance with a consensus published by a panel of experts several years ago, we do not use this terminology in the present review [29]. In the study by Huang L. et al. [30], hypoxic preconditioning increased the expression of the microglial markers Arg1, Ym1/2, IGF-1, and CD206 and reduced the expression of the markers CD16, CCL3, and iNOS despite an overall increase in the number of activated microglia. The authors also observed an increase in the levels of the anti-inflammatory cytokines IL-10 and TGF-β and a decrease in the levels of pro-inflammatory cytokines [30]. In another study using in vivo and in vitro models, hypoxic preconditioning was accompanied by reduced expression of COX-2 and iNOS as well as decreased levels of IL-1β and TNF-α [31]. A similar hypoxic preconditioning protocol, involving exposure to 7.8% O_2_ for 3 h, reduced microglial activation and decreased the expression of the inflammatory cytokines and markers TNF-α, CD11b, CD86, and IL-1β while also reducing the expression of anti-inflammatory markers and mediators such as CD206 and TGF-β [32]. In a study conducted on a mouse model of Alzheimer’s disease, intermittent hypoxia with 14.4% O_2_ for 4 h over 14–28 days reduced the levels of the pro-inflammatory cytokines IL-1β and IL-6, while it had no effect on TNF-α levels [32]. In addition, the same study showed an overall decrease in activated microglia, suggesting reduced tissue damage associated with the accumulation of amyloid β and other markers of Alzheimer’s disease [33].

One mechanism that may underlie the reduction in microglial activation is the effect on the fractalkine (FKN)/CX3CR1 axis [34]. The chemokine FKN and its receptor CX3CR1 play an important role in neuron–microglia interactions and regulate microglial activation, neuronal survival, and synaptic plasticity [35]. The FKN/CX3CR1 axis remains insufficiently studied; however, it is already known to be involved in synaptic plasticity, including plasticity mediated by microglia [35,36]. At the same time, hypoxic postconditioning has been shown to increase FKN and CX3CR1 levels in neurons, which may lead to activation of neuronal Akt signaling and prevention of cell death [34,37]. Meanwhile, hypoxic postconditioning reduces CX3CR1 expression in microglial cells [34]. Thus, hypoxic exposure under different protocols may exert multidirectional effects on the FKN/CX3CR1 axis in neurons and microglia, increasing neuronal survival while reducing microglial activation and neuroinflammation [34].

Thus, hypoxic exposure under different regimens can exert bidirectional effects on the FKN/CX3CR1 axis in neurons and microglia, enhancing neuronal survival while reducing microglial activation and neuroinflammation [34]. This finding may underlie the alterations in microglial properties described previously [30,31,32,33].

The potential role of astrocytes in neuroinflammation and neuroprotection during hypoxic conditioning deserves mention. In one classic study, researchers noted the role of astrocytes in elevating extracellular adenosine and erythropoietin levels under hypoxic conditioning, potentially exerting neuroprotective effects, whereas increased extracellular glutamate and VEGF levels may promote neuronal damage. The net impact of astrocytes on pathological processes in the CNS depends on the balance of these factors [38]. In a study conducted on cortical spheroids, hypoxic preconditioning led to astrocyte activation and reduced microglial activation. These effects were accompanied by decreased mRNA expression of C1qa and TNF-α. Such changes may be related to complex astrocyte–microglia interactions, which, under certain conditions, can reduce the severity of neuroinflammation [39]. In the aforementioned study [30], hypoxic preconditioning was observed to reduce the activation of both microglia and astrocytes. Potential differences in outcomes may be related to the different models used across studies. Hypoxic preconditioning promotes the expression of several protective factors in astrocytes, such as PGC-1α and UCP2 [Hypoxia preconditioning improves structure and function of astrocytes mitochondria via PGC-1α/HIF signal | Journal of Biosciences | Springer Nature Link]. At the same time, HIF-1α, which plays an important role under hypoxic conditions, promotes the secretion of IL-1β and pro-inflammatory chemokines (MCP-1 and MCP-5), as we will discuss later in the context of the role of HIF-1α in neuroinflammation [40]. In conclusion, the role of astrocytes in neuroinflammation and CNS injury under conditions of hypoxia and hypoxic conditioning depends on the gas exposure regimen and the balance between the pro-inflammatory properties of astrocytes—mediated in part by HIF-1α—and the anti-inflammatory properties that develop under preconditioning.

The PI3K/Akt signaling pathway plays an important role in ischemia/reperfusion injury [41]. PI3K activation occurs through interaction with receptors possessing tyrosine kinase activity and leads to Akt activation, followed by involvement of mTOR- and MYC-associated signaling components that support cell-cycle progression and protein synthesis [42,43]. HIF-1α accumulation also occurs, leading to activation of the expression of genes responsible for anaerobic glycolysis, angiogenesis, and erythropoiesis [44]. Phosphorylation of FOXO3a prevents its translocation into the nucleus and subsequent apoptosis [41]. Phosphorylation of GSK-3β by Akt also prevents apoptosis [45]. In the study by Yin et al. [44], hypoxic preconditioning, consisting of 3 h in an atmosphere containing 8% O_2_, restored the level of phosphorylated Akt and its substrate, phosphorylated GSK-3β, in neurons and microglia in an MCAo model. This led to a significant reduction in levels of pro-inflammatory markers, including NF-κB (p65), COX-2, CD68, myeloperoxidase, microglial activation, and neutrophil infiltration. In addition, proteomic analysis demonstrated suppression of nine pro-inflammatory cytokines induced by ischemic injury, including IL-1α, IL-6, IL-10, TNF-α, and MCP-1 [46]. A PI3K/Akt inhibitor blocked the restoration of phosphorylated Akt and the inactivation of GSK-3β; increased the expression of NF-κB, COX-2, and CD68; and partially abolished the neuroprotective effect by increasing the volume of brain injury [46]. In studies by Zhan L. et al., hypoxic preconditioning also activated the PI3K/Akt pathway [37,47].

Another potential mechanism underlying the anti-inflammatory and neuroprotective effects of hypoxia is the formation of exosomes after hypoxic exposure [48,49,50]. Exosomes can be released by cells under hypoxic conditions [51], e.g., from microglia exposed to oxygen–glucose deprivation (OGD), a widely accepted in vitro model of hypoxia/ischemia. Exosomes obtained from this cell culture promoted a decrease in TNF-α, iNOS, and IL-1β levels and an increase in IL-10 and CD206 levels [51]. When preconditioned exosomes were administered to animals after MCAo, a similar pattern was observed in the peri-infarct area, accompanied by corresponding changes in the cytokine and marker profiles [51]. Such exosomes may contain TGF-β1, which activates the TGF-β/Smad2/3 signaling pathway, thereby reducing the pro-inflammatory activity of microglia, as well as the molecules miR-124, miR-135a-5p, and miR-137 [52,53,54].

The development of hypoxia leads to activation of a number of molecules whose role is to adapt an organism to current and future hypoxic exposure. HIF proteins constitute one of the key groups of molecules responsible for adaptation to hypoxia. One of the most extensively studied HIF proteins is the transcription factor HIF-1α, which is hydroxylated by prolyl hydroxylase under sufficient oxygen availability conditions [55,56]. Under hypoxic conditions, HIF-1α accumulates, translocates into the nucleus, dimerizes with the β subunit, binds to the coactivators CREB and p300, and interacts with the hypoxia-responsive element, resulting in activation of the transcription of a wide range of genes involved in adaptation to hypoxia [55,56,57]. The effects of HIF-1α that support adaptation to hypoxia, including during preconditioning, include the expression of VEGF, erythropoietin, glycolytic enzymes, and glucose transporters such as GLUT-1 and GLUT-3 and other effects [2,15]. Increased HIF-1α levels predominantly promote the development of inflammation in astrocytes through the expression of IL-1β and pro-inflammatory chemokines, including MCP-1 and MCP-5 [58]. HIF also increases TLR-4 expression on the microglial cell membrane, thereby promoting microglial activation and activation of NF-κB [40]. The NLRP3 inflammasome, which plays a role in inflammation and pyroptosis, is also activated by HIF-1α [59]. At the same time, ischemic preconditioning mediated by HIF-1α’s effects was found to decrease the levels of the pro-inflammatory cytokines TNF-a, IL-1β, and IL-6 [60]. An HIF-1α inhibitor blocked these changes; however, these findings should only be extrapolated to the effects of ischemic/hypoxic preconditioning with caution since they were obtained in a model of remote preconditioning. The anti-inflammatory properties of HIF-1α may also be mediated through erythropoietin, which prevents activation of the NLRP3, NLRC4, and AIM2 inflammasomes in microglia [61]. Administration of erythropoietin before and after ischemia was found to reduce microglial activation and improve behavioral outcomes in animals [62].

Based on the foregoing information, one can conclude that HIF-1α exhibits predominantly pro-inflammatory properties in the CNS. This conclusion is supported by data derived from studies of astrocytes and microglia, as well as from investigations into the role of TLR-4 and NF-κB in their function, together with evidence on peripheral levels of pro-inflammatory cytokines [58,59,60,61,62]. Thus, hypoxia plays a dual role in the mechanisms of nervous tissue injury: prolonged, severe, or repeated hypoxia with critically low oxygen levels enhances neuroinflammation, promotes microglial and astrocyte activation, and activates NF-κB, the NLRP3 inflammasome, and pro-inflammatory cytokines, thereby aggravating the outcomes of ischemic and hypoxic brain injury. At the same time, intermittent and controlled short-term hypoxia used in pre- and postconditioning protocols exhibits pronounced neuroprotective and anti-inflammatory properties: it suppresses NF-κB and NLRP3 activation, modulates microglial and astrocyte activation, activates the protective PI3K/Akt pathway, and modulates the fractalkine/CX3CR1 axis. However, the role of HIF-1α in the development of neuroinflammation is not entirely unambiguous and may involve a predominance of pro-inflammatory properties. Thus, it is the nature, intensity, duration, and protocol of hypoxic exposure that determine whether hypoxia acts as a damaging factor or a mechanism of adaptive protection.

Synthesis of data on the effects of hypoxic pre- and postconditioning on inflammatory signaling molecules and pathways convincingly demonstrates that these interventions modulate the inflammatory process toward its suppression. This effect is achieved through the simultaneous inhibition of key pro-inflammatory cascades, including NF-κB, the NLRP3 inflammasome, and the production of pro-inflammatory cytokines, together with the enhancement of several anti-inflammatory and protective mechanisms, including IL-10 and IκB. Such complex immunomodulation is likely to represent one of the fundamental mechanisms underlying the neuroprotective action of hypoxic conditioning in ischemic brain injury. Summarized information on the effects of hypoxic conditioning on inflammatory signaling pathways is presented in Table 1.

## 4. Permissive Hypercapnia Serves as a Modulator of the Inflammatory Response: Protective Mechanisms and Effects

Permissive hypercapnia is a typical component of lung-protective ventilation strategies, in which an increase in PaCO_2_ is allowed in order to reduce ventilator-induced injury. In this context, its biological effects are not limited to the respiratory system, since hypercapnia can alter cellular signaling, inflammatory responses, and reparative processes in various tissues [63,64,65]. Publications from recent years indicate that hypercapnia should be considered a context-dependent immunomodulatory factor in inflammation rather than a universally beneficial intervention since its effects substantially depend on carbon dioxide concentration, concomitant acidosis, exposure duration, the nature of the injury, and the presence of an infectious component [64,65]. The most convincing evidence supporting the anti-inflammatory effects of hypercapnia has been obtained in models of acute sterile lung injury, endotoxemia, and ventilator-induced injury, where elevated CO_2_ was accompanied by attenuation of the NF-κB-dependent pro-inflammatory program, reduced neutrophil infiltration, and decreased severity of tissue damage [63,66,67].

One of the earliest and most reproducible mechanisms of the anti-inflammatory action of hypercapnic acidosis is its effect on the endothelial response to lipopolysaccharide. In cultured pulmonary endothelial cells, hypercapnic acidosis was found to suppress LPS-induced NF-κB activation, which was associated with reduced degradation of IκBα and attenuation of pro-inflammatory gene transcription [66]. Against this backdrop, the expression of ICAM-1 and IL-8 decreased, and neutrophil adhesion to activated endothelium was also reduced, indicating that hypercapnia affects the early vascular–inflammatory phase of injury [66]. These findings are of fundamental importance for interpreting the biological effects of CO_2_, as they show that hypercapnia can interfere not only with cytokine production but also with cellular recruitment, which determines the subsequent intensity of inflammatory tissue infiltration [63,66].

The protective effect of hypercapnic acidosis has also been confirmed at the organ level in models of endotoxin-induced lung injury [67]. In animal experiments, hypercapnic acidosis reduced the severity of acute endotoxin-induced lung injury both when applied prophylactically and therapeutically, decreasing the morphological severity of injury, impairment of gas exchange, and accumulation of reactive nitrogen-containing products in lung tissue and epithelial lining fluid [67]. A separate study showed that hypercapnic acidosis rapidly reduces oxidative reactions in an acutely injured lung in vivo, with this effect developing within minutes and not depending on peroxynitrite formation, thereby expanding our understanding of cytoprotective mechanisms under elevated CO_2_ conditions [68]. Taken together, these results allow hypercapnia to be considered a factor capable of limiting secondary tissue injury caused by excessive pro-inflammatory and oxidative activation, at least under conditions of acute sterile lung inflammation [63,67,68].

Studies on macrophages have made a substantial contribution to our understanding of the immunomodulatory action of hypercapnia since it was in these cells that the selectivity of pro-inflammatory program suppression was demonstrated [69,70]. In differentiated THP-1 macrophages, as well as in human and mouse alveolar macrophages, elevated CO_2_ levels suppressed LPS-induced production of TNF-α and IL-6, cytokines closely associated with the NF-κB-dependent innate immune response [69]. At the same time, hypercapnia did not suppress IL-10 or IFN-β, indicating that not all immune signaling pathways are completely switched off and supporting the concept of selective remodeling of the inflammatory response [69]. Critically, in this model, suppression of IL-6 was rapid, reversible, and independent of extracellular and intracellular acidosis; therefore, some immune effects of hypercapnia should be directly linked to CO_2_-dependent signaling rather than solely to a shift in pH [69,70].

This line of research was further developed in studies devoted to the stress response and the role of HSF1. It was shown that hypercapnia induced the accumulation and nuclear translocation of HSF1 in alveolar macrophages, while suppression of Hsf1 eliminated the hypercapnia-dependent reduction in IL-6 and TNF-α production in response to lipopolysaccharide administration, indicating this factor is involved in the reprogramming of inflammatory transcription [71]. In a mouse model of pneumonia caused by *P. aeruginosa*, the same mechanism was accompanied by reduced levels of IL-6, TNF-α, and IL-1β under hypercapnic conditions in animals with preserved HSF1-dependent regulation, confirming the contribution of the stress-induced transcriptional program to the immune effects of CO_2_ in vivo [71]. Thus, the molecular profile of the anti-inflammatory action of hypercapnia includes both classical nodes of NF-κB-dependent signaling and a broader stress response capable of altering the threshold and intensity of production of innate pro-inflammatory mediators [65,71].

In a similar macrophage model, elevated CO_2_ levels reduced the phagocytosis of opsonized particles and killed bacteria, thereby affecting effector mechanisms of innate immunity that are critically important for the anti-infective response [67]. At the whole-organism level, this was confirmed in a model of pneumonia caused by *P. aeruginosa*, where hypercapnia impaired neutrophil function and was accompanied by increased mortality, despite the absence of more pronounced histological inflammation in lung tissue. These results directly demonstrate a divergence between the degree of inflammatory activation and the host’s ability to mount antibacterial defense [72]. Thus, hypercapnia is appropriately characterized in contemporary reviews as a bidirectional modifier of innate immunity. On the one hand, it produces an anti-inflammatory effect and provides protection against injury under sterile conditions; on the other hand, hypercapnia may be an unfavorable factor in the presence of infection [64,65].

Considering these data, permissive hypercapnia should be described as a factor that, under conditions of acute sterile injury, can attenuate the NF-κB-dependent pro-inflammatory program, reducing ICAM-1 and IL-8 expression, decreasing TNF-α and IL-6 production, and limiting oxidative damage. However, during infection and in the reparative phase, the same mechanisms may become a source of functional losses for tissues and for the organism as a whole [66,69,72,73]. This duality is particularly important for the subsequent discussion of the effects of hypercapnia in the context of the nervous system, where direct evidence is more limited and extrapolation of findings from pulmonary endothelium and alveolar macrophages to microglia and the neurovascular unit requires separate analysis [65,74].

With regard to the nervous system, the question of the anti-inflammatory potential of permissive hypercapnia remains substantially less developed relative to the lungs, endothelium, and alveolar macrophages since the main body of evidence concerning the central nervous system relates to cerebral perfusion, apoptosis, blood–brain barrier permeability, and neurological outcomes, whereas direct studies assessing the neuroinflammatory profile, microglia, and cytokine cascades are considerably rarer [64,74]. Therefore, transferring conclusions from models of acute lung injury to the neurovascular unit necessitates particular caution because in the brain, increased PaCO_2_ levels simultaneously affect cerebral blood flow, acid–base status, barrier function, the cellular stress response, and innate immune activity. The resulting biological effect is therefore determined by the combination of these processes rather than by a single anti-inflammatory mechanism [64,75].

Nevertheless, it is precisely the cerebrovascular and anti-apoptotic effects of moderate hypercapnia that explain why it has attracted attention in neurobiology and neurocritical care [64,74]. In a model of transient global cerebral ischemia in adult rats, mild and moderate hypercapnia at PaCO_2_ levels of 60–100 mmHg improved neurological outcomes and reduced injury, whereas severe hypercapnia at PaCO_2_ levels of 100–120 mmHg aggravated cerebral edema and worsened injuries, indicating that nervous tissue had a dose-dependent response to CO_2_ [74,76]. In a model of focal ischemia followed by reperfusion, a therapeutic level of hypercapnia, defined as 80–100 mmHg of PaCO_2_ for 2 h, improved functional recovery, reduced lesion volume, and was accompanied by anti-apoptotic shifts, including increased levels of Bcl-2 and decreased levels of Bax and caspase-3. This finding is consistent with the concept of a neuroprotective window for moderate hypercapnia in acute ischemic injury [77,78]. A similar direction of effect was also observed in a traumatic model of lateral fluid percussion brain injury, where therapeutic hypercapnia reduced blood–brain barrier damage, improved neurological outcomes, and supported tissue integrity in the area of secondary injury [75].

However, these data alone do not yet allow hypercapnia to be described as a proven anti-inflammatory factor in the central nervous system, since the studies mentioned above predominantly analyze cerebral hemodynamics, apoptosis, and barrier function, whereas direct analysis of the microglial profile and cytokine architecture is considerably less developed [5,74,75,77]. Thus, in the brain, moderate hypercapnia may produce a neuroprotective effect, but this neuroprotection is not identical to an already-proven suppression of neuroinflammation. Therefore, it should be interpreted as an integrated result of vascular, metabolic, and anti-apoptotic shifts, among which the immune component remains insufficiently characterized [64,74].

Data on chronic hypercapnia are of particular interest regarding the present topic since they show that the immune response of the brain to elevated CO_2_ levels is not unidirectional [78]. In a transcriptomic study of cardiorespiratory nuclei, mild chronic hypercapnia was mainly accompanied by increased expression of immune-associated genes and activation of immune pathways, whereas moderate chronic hypercapnia, by contrast, caused a broad decrease in the expression of genes associated with the immune response and vascular function, along with inactivation of the corresponding pathways [78]. This result is of fundamental importance since it demonstrates that even within a single anatomical region of the brain, the effect of hypercapnia on the inflammatory-stress response depends on the intensity of exposure [78].

Direct evidence was also obtained in a study of microglia in respiratory chemosensitive nuclei of the brainstem during prolonged hypercapnia [79]. In this model, longer hypercapnic exposure, consisting of 10% CO_2_ in air for 30 min, increased CD86 expression in Iba1-positive cells of the ventral respiratory column, raphe nuclei, and nucleus of the solitary tract, whereas CD206 expression remained without significant changes. This result indicates a shift in microglia toward an inflammatory phenotype without a comparable increase in regulatory features [79]. In addition, in cultures of brainstem microglia, but not hippocampal microglia, hypercapnia increased IL-1β levels in the absence of increased TGF-β, and the authors directly interpreted these findings as the development of an inflammation-like phenotype in microglia of brain structures associated with respiratory regulation [79]. This is particularly important to consider since it is here where direct evidence shows that, in the central nervous system, hypercapnia may be accompanied not by suppression but rather maintenance of a pro-inflammatory microglial response, at least in some specialized brainstem centers [79]. In turn, this notion draws attention to the need for a clear definition of the localization of the pathological focus when applying hypercapnia for recovery after an ischemic stroke.

These data should also be interpreted in consideration of the possible involvement of astrocytes in the regional heterogeneity of the response to hypercapnia. In chemosensitive structures in the brainstem, astrocytes can respond to changes in CO_2_/pH and participate in the regulation of the respiratory response [80,81]. Furthermore, astrocytes are involved in CO_2_-dependent regulation of vascular tone, including Ca^2+^-, COX-1-, and PGE_2_-dependent mechanisms of hypercapnic vasodilation [82]. In the hippocampus and cortex, their contribution may be primarily related to the control of extracellular glutamate, water homeostasis, AQP4-dependent perivascular function, and astrocyte–microglia interactions [83,84,85]. However, direct evidence of how hypercapnia alters astrocytic production of cytokines and chemokines in ischemic brain tissue remains limited.

Thus, when moving from pulmonary and endothelial models to brain tissue itself, the picture becomes noticeably more complex [76,79]. Whereas under conditions of acute sterile injury outside the central nervous system, hypercapnia often reduces NF-κB-dependent pro-inflammatory activation and cellular recruitment, in regard to the brain, there are also data demonstrating the neuroprotective effect of moderate hypercapnia in ischemia and indicating pro-inflammatory microglial reactivity during prolonged CO_2_ exposure, with both groups of findings being experimentally supported [5,76,77,78,79]. Therefore, with regard to the central nervous system, hypercapnia should, at this stage, be described as a modifier of the neuroinflammatory and stress response that depends on dose, duration, and anatomical context [78,86].

The most unfavorable response is observed when hypercapnia acts not in isolation but in a context of prolonged hypoxia, and this situation is critical for neuroinflammation [86,87]. In a model of chronic hypoxemia in adult rats, the combination of 3 h hypercapnia and hypoxia caused more-severe impairment of learning and memory, more pronounced apoptosis of hippocampal neurons, and greater activation of the NLRP3 inflammasome, caspase-1, and IL-1β than hypoxia alone; thus, under these conditions, CO_2_ enhanced the inflammatory and damaging component of hypoxic stress [86]. The authors showed that hypercapnia markedly increased the expression of NLRP3, caspase-1, and IL-1β in microglia activated by hypoxia, while pharmacological inhibition of this pathway reduced neuronal apoptosis. These findings link the worsening of cognitive outcomes specifically to the microglial inflammasome component rather than only the hemodynamic consequences of hypoxia [86].

This line of research was further developed in a study showing that a similar level and duration of hypercapnia stimulated microglial pyroptosis through inhibition of mitophagy in adult rats exposed to hypoxia. Thus, elevated CO_2_ levels against the backdrop of prolonged hypoxia enhanced microglial pyroptosis and the release of IL-1β and IL-18 through impaired mitophagy and subsequent inflammasome activation [88]. Another study pertaining to the same line of research established that while hypercapnia alone may be insufficient to increase IL-1β levels, hypercapnia can exert its effects in the presence of hypoxia. Against this background, IL-1R1 and p-IRAK-1 expression increased in the cerebrovascular endothelium, while the expression of tight junction proteins, including ZO-1, occludin, and claudin-5, exhibited a decrease, which was accompanied by greater blood–brain barrier permeability [87]. This result shows that prolonged hypercapnia in combination with hypoxia can enhance IL-1β-dependent inflammatory damage at both the microglia and vascular-component-of-the-neurovascular-unit levels [86,88].

Therefore, from a practical perspective, it is methodologically imprudent to assess hypercapnia outside the context of concomitant oxygenation, the intensity of the exposure protocol, and the nature of the primary injury [86,87,89]. In acute ischemia, a moderate increase in CO_2_ levels may improve cerebral blood flow, support tissue metabolism, and reduce apoptotic damage; however, with prolonged exposure and in combination with hypoxia, the same factor may enhance ROS-dependent inflammasome activation, increase IL-1β levels, and aggravate neuronal death and cognitive deficits [82,86,87]. Accordingly, it is most appropriate to consider hypercapnia not in isolation but within a spectrum of conditions ranging from moderate therapeutic hypercapnia under controlled oxygenation to prolonged hypercapnic hypoxemia, in which different biological mechanisms already predominate [64,86].

Thus, in the central nervous system, moderate permissive hypercapnia may be accompanied by neuroprotective effects associated with improved perfusion, reduced apoptosis, and preservation of barrier function. However, direct evidence for a stable anti-inflammatory effect of CO_2_ in the brain remains limited, while during chronic exposure, especially during prolonged combination with hypoxia, hypercapnia may support pro-inflammatory microglial reactivity, enhance IL-1β-dependent cascades, and worsen injury outcomes [5,75,77,78,79,86,87].

Table 2 presents a systematization of experimental models and the main molecular and cellular effects of hypercapnia in the nervous system, including differences between acute hypercapnia, chronic hypercapnia, and hypercapnia against the backdrop of prolonged hypoxemia.

Thus, the data presented in Table 2 show that the effects of hypercapnia on the nervous system depend primarily on the exposure protocol, its duration, and its combination with chronic hypoxemia. In models of ischemic and traumatic injuries, moderate permissive hypercapnia is predominantly associated with neuroprotective changes, including reduced apoptosis, preservation of tight junction proteins, and improved neurological outcomes.

Contradictions in the literature regarding the effects of hypercapnia on the inflammatory response in the nervous system can largely be explained by the methodological heterogeneity of studies. Different studies use fundamentally different models, ranging from in vivo ischemia and traumatic brain injury to in vitro cultures of microglia and endothelial cells, while also assessing non-identical endpoints: some studies focus primarily on neurological outcome, apoptosis, and blood–brain barrier permeability [5,75,76], whereas others analyze microglial markers, inflammasome activation, and cytokine profiles [79,86,87]. Also critical is the fact that hypercapnia in these studies effectively refers to different states—acute moderate therapeutic hypercapnia, chronic hypercapnia, and prolonged hypercapnia combined with hypoxia—each of which creates a distinct biological context and may lead to opposite effects [78,79,87]. In addition, the results depend on the CO_2_ concentration employed, exposure duration, the time window relative to injury, the anatomical localization of the brain region studied, and even the regional specificity of microglia, which has been particularly clearly demonstrated for brainstem chemosensitive structures and the hippocampus [79]. Therefore, comparison of data on hypercapnia requires consideration not only of molecular markers but also of the full experimental context.

## 5. Potential Synergy in the Effects of Hypoxia and Hypercapnia on Inflammatory Processes in the Nervous System

We have published a series of studies describing the neuroprotective effects of hypoxia and hypercapnia as a means of inducing cerebral ischemic/hypoxic tolerance [7,8,9]. At the same time, the protective effects of combined hypoxia and hypercapnia were found to be superior to their effects in isolation. These effects may be mediated by a number of mechanisms, including increased HIF-1α expression, metabolic changes, modulation of apoptosis, and maintenance of blood–brain barrier integrity [11]. In addition, the results of a randomized placebo-controlled clinical trial indicated that hypercapnic hypoxia is clinically effective as a neurorehabilitation method in the acute period after a stroke [13].

However, the mechanisms underlying the neuro-rehabilitative effect of combined hypercapnia and hypoxia that are associated with the modulation of inflammation remain poorly understood. Previously, however, an increase in BDNF in ischemic nervous tissue was observed against the backdrop of a reduction in ischemic lesion volume after a two-week course of combined and isolated exposure to hypercapnia and hypoxia induced after the modeling of photoinduced focal ischemia of the rat cerebral cortex [89]. In turn, it is known that increased BDNF levels play a positive role in recovery after a stroke through the activation of anti-inflammatory effects, neurogenesis, and neuroplasticity [90,91].

One mechanism that may underlie increased BDNF expression is MAPK activation under hypercapnic exposure [92,93]. The level of BDNF expression, in turn, is likely associated with activation of adenylate cyclase and subsequent CREB phosphorylation under the influence of protein kinase A [94]. In addition, BDNF expression may increase as a result of hypercapnia-induced enhancement of MAPK activity, which initiates the CREB/BDNF signaling cascade [93,95]. MAPK activation may also occur under hypoxic exposure [96]. Thus, the combined effect of hypoxia and hypercapnia on the MAPK signaling pathway may be responsible for greater BDNF expression in nerve cells compared with their isolated effects.

In addition, it was shown that after exposure to hypercapnic hypoxia, the concentrations of S100 and NSE proteins in the blood decreased, whereas after exposure to normobaric hypoxia, only S100 levels decreased [89]. This finding may be a result of more effective recovery of nervous tissue after a stroke and/or restoration of blood–brain barrier integrity after exposure to hypercapnic hypoxia. This concept is consistent with our previous data demonstrating that the combination of hypercapnia and hypoxia leads to the smallest changes in blood–brain barrier permeability [11]. A possible mechanism underlying this effect may be the anti-inflammatory action of hypercapnia. During the development of inflammation, NF-κB is activated, which leads to increased expression of pro-inflammatory cytokines, including TNF-α, IL-6, and IL-8, and cell adhesion molecules, including ICAM-1 and VCAM-1, which make vascular walls more permeable to immune cells [97,98]. Hypercapnia reduces the activity of both the canonical and non-canonical NF-κB activation pathways, thereby decreasing the expression of pro-inflammatory cytokines and adhesion molecules [99,100]. Increased expression of heat shock proteins after hypercapnic–hypoxic exposure [101] may also lead to reduced pro-inflammatory activity of NF-κB [71]. In addition, hypercapnia can directly affect the glycolytic activity of macrophages, potentially reflecting changes in their activation statuses [102].

Another factor influencing the restoration of blood–brain barrier integrity and inhibition of neuroinflammation under hypercapnic hypoxia may be inhibition of matrix metalloproteinases by the hypoxic component of the combined exposure. For example, it has been shown that hypoxic preconditioning may reduce the activity of MMP-2 and MMP-9, which are involved in increasing blood–brain barrier permeability during ischemia–reperfusion injury [103,104,105].

It is important to emphasize that many of the data presented above were obtained though studies on peripheral cells or under specific experimental conditions, and their direct application to neuroinflammation in ischemic injury requires further in-depth verification. This is particularly relevant in view of the limited amount of direct evidence for the anti-inflammatory action of hypercapnia in the nervous system as well as data indicating that, during chronic exposure or in combination with hypoxemia, hypercapnia may enhance inflammation.

**Figure 1:**
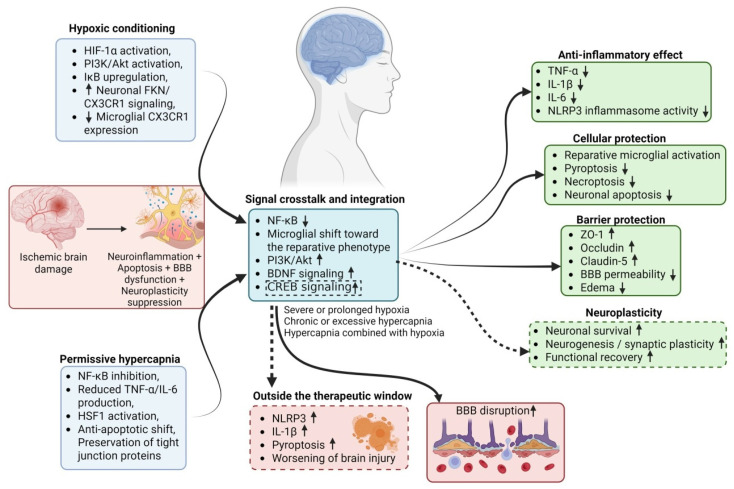
An integrative scheme of the complex protective effects of hypoxia and permissive hypercapnia on neuroinflammation and recovery processes after cerebral ischemia. Note: Hypoxic conditioning and permissive hypercapnia affect partially distinct but functionally overlapping signaling pathways, including NF-κB-dependent inflammation, microglial reactivity, PI3K/Akt-mediated survival mechanisms, CREB/BDNF-dependent neuroplasticity, and blood–brain barrier integrity. In therapeutic protocols, their combination may produce a synergistic effect manifested by attenuation of neuroinflammation, reduction in apoptosis, preservation of barrier function, and enhancement of reparative plasticity. The dashed lines indicate presumed or insufficiently verified interactions. Arrows indicate an increase (↑) or decrease (↓).

The proposed integration of hypoxic and hypercapnic signaling at the level of anti-inflammatory, barrier-protective, and neuroplastic mechanisms is shown schematically in Figure 1.

Based on the data presented in the previous sections, we can now propose a hypothesis regarding the potential mechanisms of synergistic action of moderate hypoxia and permissive hypercapnia in modulating neuroinflammation during the generation of neuroprotective effects in the CNS after an ischemic injury. Thus, hypoxic preconditioning/postconditioning and hypercapnia demonstrate a universal suppressive effect on the central pro-inflammatory transcription factor NF-κB, but through different molecular mechanisms: hypoxia increases the levels of its inhibitor IκB, preventing NF-κB translocation into the nucleus [22], while hypercapnia suppresses lipopolysaccharide-induced NF-κB activation in the endothelium and macrophages by reducing IκBα degradation [66]. Thus, the combination of these factors may create a dual blockade of NF-κB activation, leading to deeper suppression of the expression of its targets—pro-inflammatory cytokines such as TNF-α, IL-6, and IL-1β as well as adhesion molecules such as ICAM-1. It is important to emphasize that each of these factors has dose-dependent and context-specific effects, ranging from pro- to anti-inflammatory. However, their combination in optimal therapeutic protocols may lead not merely to summation but to combined effect or potential synergy that provides more pronounced and balanced neuroprotection.

Interesting observations have been made regarding the NLRP3 inflammasome: hypoxic conditioning reduced its activity and the level of phosphorylated MLKL [25,26], whereas prolonged 3 h hypercapnia against the backdrop of hypoxia, by contrast, sharply increased the expression of NLRP3, caspase-1, and IL-1β [86]. This indicates that under maladaptive prolonged hypercapnic hypoxia, a pro-inflammatory effect manifests. At the same time, a therapeutic combination of 30 min moderate hypoxia and hypercapnia may prevent this negative synergy, possibly through activation of compensatory anti-inflammatory pathways, such as induction of heat shock proteins [105].

Both factors under consideration can also affect microglial reactivity, probably through different signaling axes. For example, hypoxic conditioning promotes the expression of the microglial markers Arg1, Ym1/2, IGF-1, and CD206 as well as the anti-inflammatory cytokines IL-10 and TGF-β [30]. This effect may also be mediated by exosomes released by conditioned microglia [36]. At the same time, moderate hypercapnia may exert an anti-inflammatory effect on macrophage cells by suppressing their production of TNF-α and IL-6 [69] and influence the glycolytic metabolism of macrophage cells [102]. In this case, potential synergy may consist of hypoxia creating a signaling context, through HIF-1α and exosomes, that prepares microglia to respond to the hypercapnic stimulus, which, in turn, induces additional metabolic reprogramming of the cell toward an anti-inflammatory phenotype.

The astrocytic component must also be considered when discussing the potential interaction between hypoxic and hypercapnic signaling. The post-ischemic inflammatory response is shaped not only by microglia but also by astrocyte–microglia crosstalk: astrocytes regulate cytokine and chemokine production, extracellular glutamate levels, water homeostasis, barrier function, and the restoration of tissue homeostasis after injury [106,107]. Therefore, changes in the microglial marker profile under hypoxia or hypercapnia may reflect not only the direct action of the gas stimulus on microglia but also secondary signals emanating from reactive astrocytes.

This consideration is particularly important for hypercapnia because CO_2_ can engage astrocytic mechanisms of neurovascular regulation. In an experimental study by Howarth et al., hypercapnia was shown to induce astrocytic Ca^2+^ responses, activate COX-1, and promote PGE_2_-dependent vasodilation, indicating that astrocytes may participate in the vascular component of the brain’s response to elevated CO_2_ levels [108]. Consequently, the potential convergence of hypoxic and hypercapnic signaling should be examined not only at the microglial level but also at the level of astrocyte–microglia–neurovascular unit interactions, although direct evidence for such a mechanism under combined hypercapnic hypoxia remains insufficient.

The mechanisms of neuroprotection during combined exposure to hypercapnia and hypoxia may involve different but interconnected levels of influence on neuronal survival under conditions of neuroinflammation. In this context, hypoxia activates the PI3K/Akt pathway, leading to inhibition of the pro-apoptotic factor GSK-3β and enhancement of neuroprotection [46]. Hypercapnia also exerts anti-apoptotic effects by increasing Bcl-2 levels and reducing levels of Bax and caspase-3 [5]. Thus, combined exposure may potentiate inhibition of apoptosis under conditions of neuroinflammation, as supported by experimental data obtained from a model of ischemic injury [82].

One of the key potential convergent mechanisms of theoretical anti-inflammatory synergy or combined effect is enhanced BDNF expression [89]. Hypoxia, through activation of the p38/MAPK/MAP4 signaling pathway [96], and hypercapnia, through ERK1/2 inactivation and adenylate cyclase activation followed by CREB phosphorylation [93,94], may independently activate the MAPK/CREB pathway, leading to increased transcription of the BDNF gene. The conclusion regarding the possible convergence of the anti-inflammatory effects of hypoxia and hypercapnia at the level of the MAPK/CREB/BDNF pathway is based on data obtained from different experimental models, a fact that must be considered when further studying this effect. Their combined action may result in superactivation of this pathway, providing powerful stimulation of neuroplasticity and BDNF-mediated anti-inflammatory effects [89].

Preservation of blood–brain barrier integrity also supports the anti-inflammatory potential of both therapeutic hypercapnia, which helps reduce tissue edema [75] and preserve tight junction proteins such as ZO-1, occludin, and claudin-5 [74], and hypoxic conditioning, which prevents destruction of the blood–brain barrier basement membrane through inhibition of MMP-2 and MMP-9 [103,104]. Thus, hypoxia may prevent extracellular matrix degradation, while hypercapnia may maintain the integrity of endothelial junctions, together limiting neuroinflammation and cerebral edema.

The presented data emphasize that both hypoxia and hypercapnia exert their beneficial effects, including reducing inflammation intensity and neuroprotection, only within specific “therapeutic protocols”—under conditions of moderate intensity and limited exposure duration. Thus, moderate hypoxia in a conditioning protocol activates adaptive programs, including HIF-1α and stress proteins, while moderate hypercapnia adds anti-inflammatory and vasodilatory components without exceeding the threshold beyond which pro-inflammatory activation of inflammasomes and microglia, as well as aggravation of tissue edema, begins. By contrast, prolonged/severe hypoxia or chronic/high-level hypercapnia aggravates injuries and inflammation.

At the same time, the potential synergy between hypoxic conditioning and permissive hypercapnia in the modulation of neuroinflammation appears to be based on their complementary and multitarget effects on different components and levels of the inflammatory cascade:

- Joint suppression of the key pro-inflammatory factor NF-κB occurs at the transcriptional level;
- At the cellular level, a coordinated change in microglial activity occurs, presumably involving a transition toward a reparative phenotype;
- At the level of intracellular signaling, cross-activation of survival pathways, including PI3K/Akt, and plasticity-related pathways, including Ca^2+^/CaMKII/CREB/BDNF, is induced;
- Combined protection of the structural integrity of the blood–brain barrier and limitation of edema occur at the tissue level.

It is important to emphasize that the evidence regarding the coordinated effects on NF-κB, CREB/BDNF, blood–brain barrier integrity, and microglia has largely been derived from individual experimental studies in which these interventions were often applied separately across different models, including in vitro preparations. Further studies are required to validate the hypothesis of a possible mutual potentiation of the effects of hypoxia and hypercapnia on neuroinflammation. This potential synergy is not automatic and strictly depends on the dose parameters and duration/frequency of exposure. Further studies should be aimed at precise verification of the optimal parameters of therapeutic exposure required to achieve an anti-inflammatory effect—including the onset of exposure, gas concentrations, the duration and frequency of sessions, the repeatability of treatment courses, and other factors—as well as studying the molecular cross-points at which signals from hypoxic and hypercapnic stimuli are integrated to launch the most effective anti-inflammatory and reparative program in ischemic nervous tissue.

**Figure 2:**
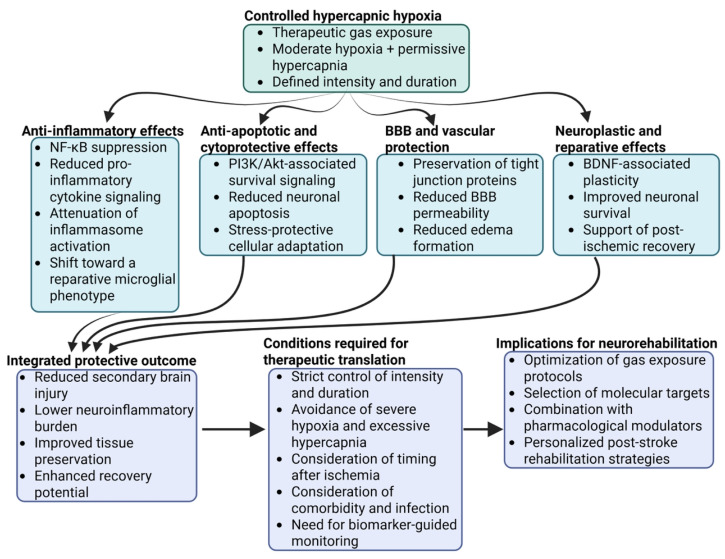
An integrative scheme of the complex protective effects of hypoxia and permissive hypercapnia on neuroinflammation and recovery processes after cerebral ischemia. Note: A controlled combination of hypoxia and permissive hypercapnia may integrate anti-inflammatory, cytoprotective, barrier-protective, and neuroplastic effects, thereby helping to reduce secondary brain injury and enhancing the potential for recovery after ischemia. The scheme also reflects the conditions necessary for the therapeutic application of this approach and the possible directions for its combination with pharmacological modulators.

The complex protective effects of a controlled combination of hypoxia and permissive hypercapnia that are relevant to post-ischemic neurorehabilitation are summarized in Figure 2.

## 6. Clinical Applications

A number of clinical studies have been conducted to evaluate the therapeutic potential of hypoxia and hypercapnic hypoxia as rehabilitation methods for patients with nervous system pathologies.

Hornby et al., in a phase II randomized trial (*n* = 35 patients with chronic stroke, duration > 6 months), evaluated the effect of combining acute intermittent hypoxia (AIH) with high-intensity walking training (HIT). The AIH protocol consisted of 15 cycles (60–90 s of 8–9% O_2_ + 30–60 s of 21% O_2_) for 30 min prior to training. The combination of AIH + HIT resulted in a statistically significant increase in maximal walking speed, six-minute-walk distance, and peak treadmill speed relative to HIT alone (*p* < 0.01) [109]. Earlier, a series of studies by Hayes et al. demonstrated the positive effect of daily intermittent hypoxia combined with walking training on walking speed and endurance in patients with chronic motor impairments, including post-stroke patients [110].

The most well-studied form of preconditioning in clinical practice remains to be remote ischemic preconditioning (RIPC), which is implemented through repeated cycles of ischemia–reperfusion in a limb using a cuff. In a large multicenter randomized RICAMIS trial (*n* = 1893 patients who suffered an moderate acute ischemic stroke), the addition of RIPC (5 cycles of 5 min) to standard therapy led to a significant increase in the proportion of patients with an excellent functional outcome (mRS 0–1) at day 90 (67.4% vs. 62.0% in the control group, *p* = 0.02) [111]. Subsequent studies (RESIST, REMOTE-CAT) confirmed the safety of RIPC when applied in both inpatient and prehospital settings, including in combination with thrombolysis and mechanical thrombectomy. Although not all large trials achieved statistical significance for the primary endpoint, this method demonstrates potential benefits and good tolerability [112,113].

One study using hypercapnic hypoxia as a neuro-rehabilitative intervention was conducted as a randomized, triple-blind, placebo-controlled trial involving 102 patients affected by an ischemic stroke [10]. Treatment was initiated 48–72 h after the onset of the stroke. The experimental group (*n* = 50) underwent daily breathing training sessions in which hypercapnic hypoxia was induced, with FetCO_2_ = 5–6% and FetO_2_ = 15–16%, consisting of 7–11 sessions of 20 min each. The control group (*n* = 52) performed similar breathing exercises with atmospheric air. According to the results of the study, the hypercapnic hypoxia group showed a statistically significant improvement in neurological status according to the NIHSS, mRS, Barthel Index, Rivermead Mobility Index, and MoCA scales as well as reduced levels of anxiety and depression.

However, neuroimaging techniques were not used in this study to reliably assess changes in stroke volume in response to the therapy applied. Furthermore, the relatively small sample size should be noted. The authors concluded that this method may represent a safe and effective approach to the early neurorehabilitation of patients after an ischemic stroke, warranting further investigation, including in clinical trials [10]. A similar approach had previously been successfully tested in regard to cerebral palsy [114]. In a randomized, triple-blind, placebo-controlled study involving 42 children aged 3–7 with spastic cerebral palsy, a course of breathing training with hypercapnic hypoxia led to substantial improvement in the functional state of the nervous system, manifested by accelerated conduction of excitation along the pyramidal tract, increased excitability of cortical motor neurons, and reduced latency of cognitive evoked potentials. It should be noted that in this study, the conclusions were based primarily on neurophysiological measures, limiting extrapolation of the results to patient clinical outcomes [114].

Future clinical studies may focus on determining the optimal regimens and duration of respiratory exposure as well as identifying patient subgroups that are more likely to benefit from such exposure. Furthermore, the long-term effects of hypercapnic hypoxia on stroke outcomes have not been established. Additionally, potential adverse events associated with this method should be mentioned, which may include increased systemic blood pressure, development of cerebral edema, and exacerbation of ischemia due to blood flow redistribution. Patient groups that may be negatively affected by hypercapnic hypoxia include those with pulmonary or cardiac pathologies. These hypotheses require validation in future studies.

## 7. Perspectives on the Pharmacological Modulation of Hypoxic and Hypercapnic Conditioning

As mentioned earlier, there are several main mechanisms that may underlie the possible synergy between hypoxia and hypercapnia in the context of neuroinflammation modulation. In addition to providing a theoretical rationale for the use of hypoxia and hypercapnia in nervous system pathology, these mechanisms may also serve as targets for pharmacological potentiation. This direction may become a task for future research.

One promising target for pharmacological potentiation is the NLRP3 inflammasome, whose important role in nervous system pathology and neuroinflammation was described in the previous sections [22,115,116]. For example, the selective NLRP3 inhibitor MCC950 significantly reduced levels of the pro-inflammatory cytokines TNF-α, IL-1β, and IL-6 and decreased the severity of neurological deficits in models of subarachnoid hemorrhage and stroke [117,118]. Hypoxia and hypercapnia may exert multidirectional effects on NLRP3: hypoxia reduces NLRP3 activity, whereas hypercapnia, in contrast, may increase its activity and promote neuroinflammation during prolonged exposure [22,85]. It is reasonable to assume that NLRP3 blockade may additionally enhance the anti-inflammatory effect of hypoxia while simultaneously reducing the potential activating influence of hypercapnia on NLRP3, which, together, may lead to reduced neuroinflammation.

Hypoxic conditioning already activates the protective PI3K/Akt signaling pathway to a considerable extent, leading to phosphorylation of GSK-3β; inhibition of pro-apoptotic factors; reduced expression of pro-inflammatory markers such as NF-κB, COX-2, and CD68; and limitation of neuroinflammation and apoptosis in models of ischemic brain injury [46]. In the context of hypercapnic hypoxia, this pathway is of particular interest since combined exposure to hypoxia and hypercapnia may potentiate PI3K/Akt activation, thereby enhancing anti-apoptotic and neuroprotective effects relative to isolated exposure.

One promising PI3K/Akt-activating molecule is SC79 [119], which specifically activates Akt in the cytoplasm by inhibiting its membrane translocation. SC79 penetrates the blood–brain barrier well and has high selectivity. In a rat model of focal cerebral ischemia/reperfusion, administration of SC79 significantly reduced stroke volume [119]. SC79 significantly increased the level of phosphorylated Akt, increased expression of the anti-apoptotic protein Bcl-2, reduced expression of the pro-apoptotic protein Bax, decreased infarct volume, and improved neurological outcomes. These effects were completely blocked by co-administration of the PI3K inhibitor LY294002, confirming that neuroprotection was mediated specifically through activation of PI3K/Akt signaling [119]. The potential synergy with the effects of hypercapnic hypoxia is evident in the fact that hypercapnic hypoxia creates a favorable background for PI3K/Akt activation through its hypoxic component and for apoptosis inhibition through its hypercapnic component, whereas SC79 provides additional, more stable, and prolonged phosphorylation of Akt in the cytoplasm. Such a combination may allow deeper inhibition of apoptosis and neuroinflammation.

Another method of pharmacologically augmenting hypercapnic hypoxia may involve targeting c-Jun N-terminal kinase (JNK). JNK belongs to the MAPK family and is activated by ischemia, oxidative stress, and other stimuli. Activation of JNK after cerebral ischemia/reperfusion leads to caspase activation, cytochrome C release, and phosphorylation of Bad at serine-128, thereby promoting apoptosis and neuroinflammation [120]. The JNK blocker IQ-1S was found to reduce inflammatory cytokine levels after lipopolysaccharide administration in mice [121]. The drug also suppressed macrophage polarization toward the proinflammatory phenotype, which may be interpreted as an anti-inflammatory effect. In another study, the lithium salt of the same compound, IQ-1L, also reduced stroke volume and levels of IL-1α, IL-1β, IL-6, GM-CSF, and MCP1 compared with the control group [122]. In a middle-cerebral-artery occlusion model (MCAo), administration of the JNK inhibitor JNK-IN-8 reduced the severity of neurological deficits; decreased IL-1β, IL-6, and TNF-α levels; and suppressed NF-κB activation [123].

There is evidence of increased BDNF levels in ischemic tissue after therapeutic exposure to hypercapnic hypoxia [89]. The use of TrkB agonists that interact with the BDNF receptor may additionally potentiate the neuroprotective and anti-inflammatory effects of hypercapnic hypoxia. The most studied candidates are 7,8-dihydroxyflavone (7,8-DHF) and LM22A-4. 7,8-DHF is a highly specific TrkB agonist that penetrates the blood–brain barrier [124]. In models of ischemic stroke and perinatal hypoxia–ischemia, it reduces infarct volume, improves motor and cognitive recovery, and enhances myelination [125]. In in vitro models, 7,8-DHF increased neuronal survival and suppressed apoptosis during oxygen–glucose deprivation [120]. The partial TrkB agonist LM22A-4 increased TrkB and Akt phosphorylation, stimulated neurogenesis, and improved functional outcome in models of acute ischemic stroke and traumatic brain injury [126].

The potential avenues for pharmacological modulation of the effects of hypoxia and hypercapnia outlined above are encouraging and promising, yet at the same time remain largely preliminary. Further studies are required in which the effects of gas exposure alone are directly compared with those of gas exposure combined with a pharmacological agent.

## 8. Conclusions

The presented analysis confirms that controlled intermittent hypoxia and permissive hypercapnia are capable of modulating key mechanisms of neuroinflammation (NF-κB, NLRP3 inflammasome, microglial functional states) and promoting neuroplasticity (CREB/BDNF). However, the evidence regarding their combined effects on neuroinflammation remains limited. The potential synergistic or mutually potentiating action of hypoxia and hypercapnia on neuroinflammatory pathways has not yet been adequately characterized. Further studies are required to validate the hypothesis that their combination exerts superior anti-inflammatory and cytoprotective effects compared with either stimulus alone. In particular, additional research is needed to determine whether targeted pharmacotherapy combined with gas conditioning offers clinical advantages over gas exposure alone. Thus, hypoxia, hypercapnia, and their combination represent promising directions that require further investigation.

## Figures and Tables

**Table 1:** Molecular effects of hypoxic conditioning on inflammatory signaling pathways.

Molecule	Description	Role in Inflammation	Changes During Pre-/Postconditioning	Reference
HIF-1α	HIF-1α is a hypoxia-activated factor. It ensures adaptation of tissue to hypoxia through angiogenesis, metabolic changes, and erythropoietin production. It is mainly considered a pro-inflammatory molecule.	↑	↑	[58,59,60]
IL-1β	IL-1β plays a role in the binding and activation of IL-1R1, leading to the activation of NF-κB and MAPK and increased synthesis and secretion of other pro-inflammatory molecules, such as IL-8, IL-6, C-C motif chemokines, TNF-α, NO, and MMPs.	↑	**↓**	[31,51]
IL-1R1	IL-1R1 is a receptor for IL-1β and a transmembrane glycoprotein. It contains three immunoglobulin-like domains in the extracellular segment as well as transmembrane and cytoplasmic domains. It regulates NF-κB and MAPK.	↑	**↓**	[26]
IL-6	This molecule binds to IL-6R and gp130, leading to cell proliferation or apoptosis.	↑	**↓**	[44,46,60]
TNFα	TNFα is a cytokine that interacts with TNFR1 and TNFR2, leading to the activation of intracellular pathways such as NF-κB and MAPK, which regulate proliferation, apoptosis, and intercellular interactions.	**↑**	**↑↓**	[31,32,33,46,51]
NF-κB	NF-κB is a group of intracellular signaling molecules involved in the canonical signaling pathway associated with the development of inflammation and immune responses. It is also involved in the non-canonical NF-κB activation pathway, which regulates lymphocyte function.	↑	**↓**	[22,46]
NLRP3	This molecule is a component of the inflammasome, which activates IL-1 and IL-18 through caspase-1.	↑	**↓**	[22,25]
MLKL	MLKL is a pseudokinase lacking phosphorylation activity. It forms pores that increase cell membrane permeability, leading to necroptosis. It also affects other intracellular pathways, such as NF-κB.	↑	**↓**	[22,26]
COX-2	COX-2 is an isoform of COX that converts arachidonic acid into prostaglandins. It may be involved in the pathogenesis of neurodegenerative diseases.	↑	**↓**	[31,46]
iNOS	iNOS is an isoform of NOS that converts arginine into citrulline and NO. It may play a role in the development of CNS pathology.	↑	**↓**	[30,31,51]
FKN (CX3CL1)	This molecule is a ligand of the CX3CR1 receptor and a chemokine. It regulates cell adhesion, chemotaxis, and inflammation. In the nervous system, it also affects neuroplasticity and neuronal survival.	↑↓	↑ neurons, ↓ microglia	[34,37]
CD11b	CD11b is an integrin located on the surfaces of macrophages and granulocytes, where it mediates cell adhesion, migration, and phagocytosis.	↑	↓	[32]
CD86	CD86 provides co-stimulation during the interaction between antigen-presenting cells and T lymphocytes.	↑	↓	[32]
CD206	This molecule is a pattern-recognition receptor that binds to mannose residues and mediates phagocytosis and endocytosis in macrophages.	↑	↓	[30,32,51]
TGF-β	TGF-β is a growth factor that regulates inflammation, tissue repair, angiogenesis, and fibroblast function.	↑↓	↑↓	[30,32]
IL-10	IL-10 is an anti-inflammatory cytokine that reduces NF-κB activity and regulates the activity of the JAK–STAT pathway. It decreases the expression of Th1 molecules and macrophage-costimulatory molecules.	↑	↑	[30]
IκB	This molecule prevents phosphorylation of the NF-κB dimer and its translocation into the nucleus, thereby preventing the transcription of pro-inflammatory cytokines.	↓	↑	[22]

Note for Table 1: Arrows indicate an increase (↑) or decrease (↓) in inflammatory activity under the influence of the molecules listed in the table.

**Table 2:** The molecular effects of hypercapnia in the nervous system.

Type of Hypercapnia	Conditions/Model (CO_2_, Duration)	pH	Localization/Object	Main Effects	Biological Interpretation	Reference
Mild/moderate, permissive, acute	Transient global cerebral ischemia in rats; 2 h reperfusion; PaCO_2_ 60–80 or 80–100 mmHg	7.21 ± 0.07 (PaCO_2_ 60–80); 7.13 ± 0.09 (PaCO_2_ 80–100)	Brain; tissue level	Improved neurological outcome; reduced brain injury	Neuroprotective window for moderate hypercapnia	[76]
Severe, permissive, acute	Same model; 2 h reperfusion; PaCO_2_ 100–120 mmHg.	7.05 ± 0.10	Brain; tissue level	Increased cerebral edema; worsening of injury	Loss of the protective effect at a high degree of hypercapnia	[76]
Moderate, permissive/therapeutic, acute	Focal ischemia–reperfusion (MCAO/R) in rats; after 90 min of ischemia; PaCO_2_ 80–100 mmHg for 2 h	Not stated	Ischemic cortex; neuronal/tissue level	Bcl-2 ↑; Bax ↓; caspase-3 ↓; TUNEL-positive neurons ↓; infarct volume ↓	Anti-apoptotic shift; improved functional recovery	[5]
Moderate, permissive/therapeutic, acute	Lateral fluid percussion brain injury; PaCO_2_ 80–100 mmHg; assessment after 3 h	7.05–7.15 (CO_2_ for 3 h)	Perifocal zone; endothelium; BBB	ZO-1 ↑; occludin ↑; claudin-5 ↑; BBB permeability ↓	Maintenance of tight junctions; limitation of secondary barrier damage	[75]
Chronic, mild	Female goats; 6% inhaled CO_2_ for 14 days; approximately PaCO_2_ ~55 mmHg	Not stated	Cardiorespiratory nuclei of the brainstem	Activation of immune-associated genes and pathways	Maintenance of neuroinflammatory/immune activity under mild chronic hypercapnia	[78]
Chronic, moderate	Female goats; 6% CO_2_ for 7 days + 8% CO_2_ for another 7 days; approximately PaCO_2_ ~65 mmHg	Not stated	Same cardiorespiratory nuclei	Reduced expression of genes associated with the immune response and vascular function	Inactivation of immune and vascular pathways under more pronounced chronic hypercapnia	[78]
Acute, experimental, region-specific	Mice; 10% CO_2_ for 30 min	Not stated	Ventral respiratory column, raphe nuclei, nucleus of the solitary tract; microglia	CD86 ↑; CD206—no significant shift; morphological reactivity of microglia	Shift toward reactive/pro-inflammatory microglia in chemosensitive nuclei of the brainstem	[79]
Acute, experimental, in vitro	Primary microglial cultures; 10% CO_2_; pH 7.2; 2 h exposure + 22 h recovery	pH 7.2 during hypercapnic exposure; pH 7.4 before exposure and during the recovery period.	Brainstem microglia vs. hippocampal microglia	IL-1β ↑ in brainstem microglia; TGF-β—no significant shift; no pronounced increase in IL-1β in the hippocampus	Regional heterogeneity of the microglial response to hypercapnia	[79]
Prolonged against the backdrop of hypoxia; mixed in vivo/in vitro model	In vivo: 3 h ventilation, 16% O_2_ + 5% CO_2_; in vitro: 0.2% O_2_ + 15% CO_2_	7.20–7.25 (in vivo and in vitro)	Hippocampus; hypoxia-activated microglia	NLRP3 ↑; caspase-1 ↑; IL-1β ↑; increased neuronal apoptosis; cognitive deficit	Inflammasome activation and enhancement of neuroinflammation when CO_2_ is combined with hypoxia	[86]
Prolonged against the backdrop of hypoxia; mixed in vivo/in vitro model	In vivo: 3 h ventilation, 16% O_2_ + 5% CO_2_, PaO_2_ 55–60 mmHg, pH 7.20–7.25; in vitro: 0.2% O_2_ + 15% CO_2_; early time points after ventilation; exposure for 3 h	7.20–7.25 (arterial blood and in vitro).	Nervous tissue; microglia	Caspase-1 ↑; IL-1β ↑; IL-18 ↑; ROS ↑; signs of mitophagy suppression	Pyroptotic response of microglia; enhancement of neuroinflammation under impaired mitophagy	[88]

Note for Table 2: For in vivo models, arterial blood pH is indicated; for in vitro models, the pH of the culture medium or supernatant is indicated (when reported by the authors). For studies that did not describe the pH of the experimental environment, the value “not reported” is given in the corresponding column; therefore, effects attributable to hypercapnia per se versus those mediated by acidosis should be interpreted with caution. Arrows denote upregulation (↑) or downregulation (↓) of the activity or abundance of molecules, as well as alterations in the state of structures, mediated by the hypercapnic regimens specified in the table.

## Data Availability

No new data were created or analyzed in this study.
